# Mechanistic link between right prefrontal cortical activity and anxious arousal revealed using transcranial magnetic stimulation in healthy subjects

**DOI:** 10.1038/s41386-019-0583-5

**Published:** 2019-12-02

**Authors:** Nicholas L. Balderston, Emily M. Beydler, Camille Roberts, Zhi-De Deng, Thomas Radman, Tiffany Lago, Bruce Luber, Sarah H. Lisanby, Monique Ernst, Christian Grillon

**Affiliations:** 1 Section on Neurobiology of Fear and Anxiety, National Institute of Mental Health, National Institutes of Health, Bethesda, MD USA; 2 Center for Neuromodulation in Depression and Stress, Department of Psychiatry, University of Pennsylvania, Philadelphia, PA USA; 3 Noninvasive Neuromodulation Unit, National Institute of Mental Health, National Institutes of Health, Bethesda, MD USA

**Keywords:** Cognitive neuroscience, Anxiety

## Abstract

Much of the mechanistic research on anxiety focuses on subcortical structures such as the amygdala; however, less is known about the distributed cortical circuit that also contributes to anxiety expression. One way to learn about this circuit is to probe candidate regions using transcranial magnetic stimulation (TMS). In this study, we tested the involvement of the dorsolateral prefrontal cortex (dlPFC), in anxiety expression using 10 Hz repetitive TMS (rTMS). In a within-subject, crossover experiment, the study measured anxiety in healthy subjects before and after a session of 10 Hz rTMS to the right dorsolateral prefrontal cortex (dlPFC). It used threat of predictable and unpredictable shock to induce anxiety and anxiety potentiated startle to assess anxiety. Counter to our hypotheses, results showed an increase in anxiety-potentiated startle following active but not sham rTMS. These results suggest a mechanistic link between right dlPFC activity and physiological anxiety expression. This result supports current models of prefrontal asymmetry in affect, and lays the groundwork for further exploration into the cortical mechanisms mediating anxiety, which may lead to novel anxiety treatments.

## Introduction

The bulk of the mechanistic research into anxiety expression implicates sub-cortical structures such as the amygdala and bed nucleus of the stria terminalis; [1] however, there exists a large body of literature implicating large-scale brain networks in anxiety [2]. In addition, individuals with anxiety disorders exhibit wide-ranging symptoms [3] that likely involve distributed neural circuits with multiple regions contributing to expression [4]. By broadening our understanding of the mechanisms mediating anxiety expression, it may be possible to develop new treatments for anxiety disorders. One potential approach toward this goal is to use transcranial magnetic stimulation (TMS), which can directly activate cortical neurons via ultra-brief local magnetic field changes, to probe candidate regions in the networks thought to be important for anxiety. The general purpose of this study is to use rTMS to probe one specific candidate region, the dorsolateral prefrontal cortex (dlPFC), and determine the role of this region in anxiety expression.

We chose the dlPFC because previous studies indicate that this region may be important for top-down regulation [5], which may be important for anxiety [6, 7]. For instance, BOLD responses in the dlPFC during threat are negatively correlated with subjective anxiety [6], tasks that activate the dlPFC reduce anxiety potentiated startle (APS), and dlPFC activity during threat positively correlates with performance when task demands are high [7]. Together these results suggest that facilitating dlPFC activity should reduce anxiety; however, this is not reflected in the current therapeutic application of rTMS to treat anxiety symptoms in depression [8]. Rather than facilitating dlPFC activity, these clinical rTMS protocols are designed to reduce dlPFC excitability in the right hemisphere. This type of application is consistent with the interpretation that the right dlPFC is important for anxiety expression rather than regulation [9], but inconsistent with our previous results.

Accordingly, to distinguish between these two possibilities, we targeted this region with 10 Hz rTMS, based on previous results showing excitatory effects with high-frequency (>5 Hz) stimulation [10]. Because brain state at the time of stimulation influences response to the stimulation [11], we delivered the stimulation during the maintenance interval of the Sternberg working memory paradigm, a task known to activate the dlPFC [12], to facilitate the effectiveness of this stimulation. We also used this task to identify individualized functional TMS targets, representing the peak BOLD activity during the maintenance interval in the dlPFC for each subject, and used iterative electric-field modeling to optimize the coil position [13].

To induce aversive states in our subjects, we used the NPU (Neutral, Predictable, and Unpredictable) threat task, a well-validated and robust way to evoke acute fear and sustained anxiety [14]. The NPU threat task uses both predictable and unpredictable threats of shock to probe acute fear and sustained anxiety responses within subject, respectively [15]. Fear and anxiety were measured using fear- and anxiety-potentiated startle (FPS, and APS, respectively), the change in the magnitude of the startle reflex during predictable and unpredictable threat periods compared to safe periods. Potentiated startle has been extensively researched [16, 17], and shown to be reliable across sessions [18].

**Fig. 1:**
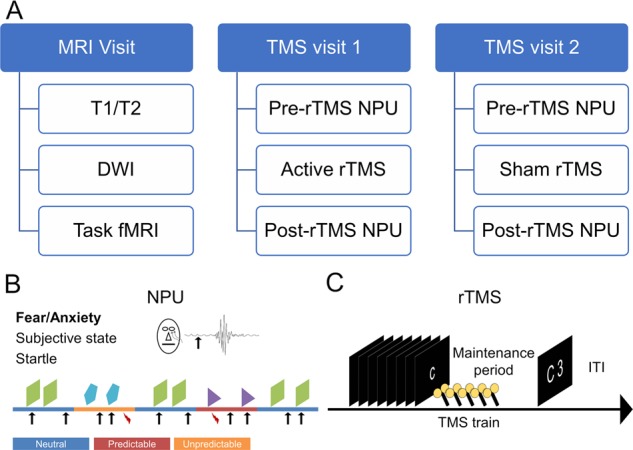
Overall design of the experiment. **a** Overall design of the study. We tested anxiety before and after rTMS using the Neutral, Predictable, and Unpredictable (NPU) threat task. **b** NPU design: during the neutral blocks, subjects were not at risk for receiving a shock. During the predictable blocks, subjects were at risk for receiving a shock only when cued by a shape presented on the screen. During the unpredictable blocks, subjects were at risk for receiving a shock throughout the entire block. Arrows indicate startle probes, lightning indicates shock presentations. **c** Sternberg Working Memory (WM) paradigm design: subjects were then presented sequentially with a series of either 5 or 8 letters. On sort trials (5 letters only), subjects were asked to rearrange the letters in alphabetical order. On maintain trials (low = 5 letters; high = 8 letters), subjects were asked to remember the letters without rearrangement. After a brief delay interval, subjects were presented with a letter and a number, and asked to indicate with a forced-choice button press whether the position of the letter in the series matched the number. TMS coils indicate timing of the TMS trains.

The specific objective of the study then, was to examine the effect of high-frequency rTMS to the dlPFC on sustained anxiety (See Fig. 1). We chose the right dlPFC based on previous data from our lab indicating a potential link between this region and anxiety regulation [6]. We administered active or sham 10 Hz rTMS to the right dlPFC on separate days, and measured the effect of this rTMS on fear and anxiety using the NPU task. Given the role of the dlPFC in anxiety regulation [5], we specifically focused on sustained anxiety during the unpredictable periods. We chose this pre-post design to minimize the off-target effects of aversive sensation of the prefrontal rTMS delivery. We assessed target the ability of the TMS to influence prefrontal circuits (i.e. target engagement) by examining performance on the Sternberg WM paradigm during the TMS administration. We hypothesized that subjects would show reduced APS following active but not sham stimulation.

## Materials and methods

### Participants

Twenty-four participants were recruited from the Washington, DC, metropolitan area to take part in this study (See Consort Flowchart in Supplement). Exclusion criteria included: current or past Axis I psychiatric disorder(s) as identified with the Structured Clinical Interview for DSM-IV, non-patient edition [19], use of psychoactive medications, any significant medical or neurological problems (e.g. cardiovascular illness, respiratory illness, neurological illness, seizure, etc.), and any MRI/TMS contraindications (e.g. implanted metal, history of epilepsy or seizure, etc.). For a complete list, see: www.clinicaltrial.gov (Identifier: NCT03027414). Five subjects did not complete the study for the following reasons: discomfort associated with TMS (2 subjects), scheduling conflict (1 subject), incompatible hairstyle (1 subject), dizziness (1 subject) leaving 19 subjects (10 females, mean age = 28.42 years, SD = 8.15). All participants gave written informed consent approved by the National Institute of Mental Health (NIMH) Combined Neuroscience Institutional Review Board and were compensated for their time.

### Procedure

The study used a within-subject, crossover design where subjects completed an MRI and 2 TMS study visits. During the MRI visit, Subjects were consented and screened for MRI/TMS contraindications [20]. They then completed a T1, T2, a diffusion-weighted EPI scan, and a 10-m resting state scan, followed by two ~8 m EPI scans during which they performed the Sternberg WM task. Scanning took place in a Siemens 3T Skyra MRI scanner with a 32-channel head coil, with a coil mounted mirror system for visual stimulus delivery. The data from the MRI visit were used to localize the optimal stimulation site and calculate the e-field models [21]. Testing took place during the TMS visits where subjects’ anxiety was assessed using the NPU threat task before and after 10 Hz stimulation to the right dlPFC. Subjects completed the following procedures: electrode setup, startle habituation, shock workup, pre-stimulation NPU, motor thresholding, TMS administration during the Sternberg WM task, and post-stimulation NPU. Active and sham stimulation were delivered on separate days, and the order was counterbalanced across subjects according to a random number sequence generated by the research assistants.

### Sample size determination

We conducted a power analysis based on data from a pilot study that used the Sternberg working memory task to reduce APS [22]. In our Sternberg pilot experiment, we obtained the effect size (*f* = 0.72) for our observed WM load-related decrease in anxiety. Assuming a somewhat smaller effect size (*f* = 0.5) due to regression to the mean, we set power at 0.80 and experiment-wise, two-tailed alpha at 0.05. Based on these parameters, we estimated that we would need 26 subjects.

### Sternberg working memory task

On each trial, subjects were sequentially presented a series of 5 or 8 letters, followed by a brief delay period [22]. On “maintain” trials (low = 5 letters; high = 8 letters), subjects rehearsed the series in order. On “sort” trials (5 letters), subjects rearranged the letters in alphabetical order. At the response prompt, subjects were presented with a letter and a number, and asked to indicate with a button press whether the position of the letter in the series matched the number. During the MRI session, there were 48 total trials (half match, half mismatch). During the TMS session, there were 42 total trials, which were nested within the 10 Hz stimulation protocol, such that the TMS trains always occurred during the WM maintenance interval [23]. The duration of the letter series (2.5–5.5 s), the delay interval (2.5–6.5 s), and the ITI (3–8 s) were jittered the trial onset cue and the response prompt were presented for 1 and 3 s, respectively.

### NPU threat task

Subjects were presented with neutral (no shock), predictable (at risk for shock only during cue), and unpredictable blocks (at risk for shock at all times) [15]. White noise probes were presented every ~17 s (jittered). Half of the probes were presented during the cue, half were presented during the ITI. Cues were (8 s) simple colored (orange, teal, and purple) geometric shapes (triangle, square, and pentagon), and color/shape were randomly assigned to conditions. Three shocks were presented in each run at a random point during either the cue (predictable condition) or the ITI (unpredictable condition). Subjects were instructed to rate their anxiety from 0 (not anxious) to 10 (extremely anxious) via an onscreen scale present for the duration of the task.

### Scans

Scanning took place in a Siemens 3T Skyra MRI scanner with a 32-channel head coil, with a coil mounted mirror system for visual stimulus delivery. We acquired a T1-weighted MPRAGE (TR = 2400 ms; TE = 2.24 ms; flip angle = 7°) with 176, 0.8 mm axial slices (matrix = 256 mm × 256 mm; field of view (FOV) = 204.8 mm × 204.8 mm). We acquired a T2-weighted image (TR = 3200 ms; TE = 566 ms; flip angle = 120°) with 208, 0.8 mm sagittal slices (matrix = 300 mm × 320 mm; FOV = 240 mm × 256 mm). We also acquired a diffusion-weighted image (TR = 12000 ms; TE = 94 ms; flip angle = 90°, B0 = 1000) with 70, 2.0 mm axial slices (matrix = 128 mm × 128 mm; FOV = 256 mm × 256 mm) aligned to the AC-PC line and 30 directions. Finally, for each multi-echo BOLD scan we acquired whole-brain images (TR = 2000 ms; TEs = 13.8, 31.2, 48.6 ms; flip angle = 70°) comprised of 32, 3 mm axial slices (matrix = 64 mm × 64 mm; FOV = 192 mm × 192 mm) aligned to the AC-PC line. For the resting state scan, we acquired 300 BOLD images. For each of the task-based scans, we acquired 230 BOLD images. In addition, we acquired a 10 BOLD reverse phase-encoded “blip” image to correct for geometric distortion in the EPI data.

### fMRI Pre-processing

Preprocessing was done with the AFNI meica.py script [24], which included slice-timing correction, despiking, volume registration, TE-dependent independent components analysis (ICA) denoising, scaling, EPI distortion correction, and blurring with a 6-mm FWHM gaussian kernel. Timeseries were then scrubbed for motion >0.5 mm RMS. First level GLM modeling included variable duration blocks for the letter series, maintenance interval, and response prompt [25]. It also included regressors of no interest corresponding to the 6 motion parameters and 4 polynomial baseline estimates.

### Target localization

**Fig. 2:**
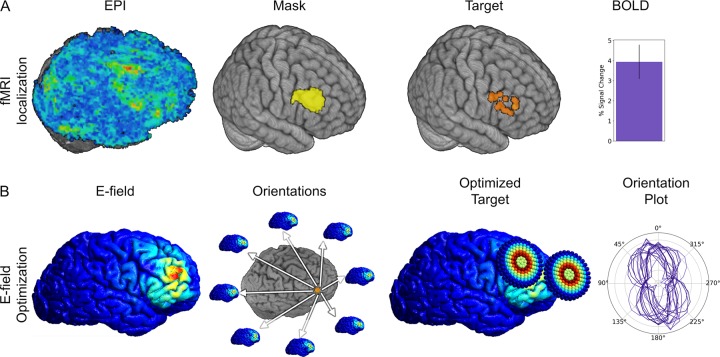
fMRI data and Electric-field (E-field) models used for target localization. **a** Pipeline for fMRI localization EPI maps are created for the sort > low contrast. These were sampled using a group-level mask. The peak within the mask was extracted and used as the TMS target (figure shows targets used for all subjects). BOLD activity was extracted from targets and averaged across subjects. Bar indicates Mean ± SEM. **b** E-field models were calculated at the target for each subject. This was repeated across 24 equally spaced coil handle orientations. The optimized target was defined as the coil handle orientation that generated the largest e-field at the target location. An orientation plot was generated showing the e-field amplitude plotted as a function of the coil handle orientation for each subject. Lines correspond to single-subject e-field model amplitudes.

We used WM-related activation contrasts (sort > low) to define the X, Y, and Z target coordinates for each subject. We identified the BOLD peak within the right dlPFC, which was defined using a group-level functional ROI from a previous study using the same Sternberg WM task (See Fig. 2a) aligned to native space.

### Electric-field optimization

We used electric-field modeling to define the roll, pitch, and yaw vectors of the TMS coil for each subject during stimulation (See Fig. 2b) [13]. Tissue compartments were created from the T1/T2 images for the skin, skull, cerebrospinal fluid, gray matter, and white matter using SimNIBS [21]. Conductivity tensors were then created from the DWI images for the with matter compartment. Next, the electric-field in these compartments was modeled in a series of 24 independent simulations, corresponding to a coil position with the target XYZ coordinates, roll and pitch vectors tangent to the scalp, and 24 evenly spaced yaw vectors [26]. The simulation with the largest normalized electric field strength estimate in the dlPFC gray matter was used to define the yaw vector.

### Neuronavigation

We used the Brainsight (Rogue Research Inc, Montreal, Canada) frameless stereotaxic neuronavigation system for neuronavigation. Subjects were registered to their T1 image and target via fiducial points at the nasion and tragi. Subject and coil position were tracked in real-time via reflective markers monitored with an infrared camera setup, and corrected for subject movement as needed. In addition, the position of the coil relative to the target was logged at the start of each TMS train.

### Motor threshold determination

Resting motor threshold (MT) was defined as the minimum magnetic flux needed to elicit a threshold motor evoked potential (MEP) ≥50 µV in the first dorsal interosseus (FDI) muscle in 5 out of 10 trials [27, 28].

### Active rTMS

A Magventure MagPro 100 (MagVenture, Inc., Alpharetta GA) stimulator equipped with a Cool-B65 A/P coil was used to deliver the rTMS to the right dlPFC target defined above. Subjects received a total of 42, four-second trains of 10 Hz stimulation during the WM delay period (jittered 3–5 s after delay onset) starting at 100% MT. Intensity was lowered upon request of the subject to avoid non-specific effects of the sensation of the TMS pulse on subjects’ anxiety. Subjects’ final intensity was ~90% MT, and did not differ between active and sham (Active: *M* = 89.0 %MT, SD = 13.5; Sham: *M* = 91.6 %MT, SD = 13.0; *p* > 0.05). Similarly, subjects’ intensity ratings (1[not unpleasant]−10 [extremely unpleasant]) did not differ between active and sham (Active: *M* = 6.3, SD = 2.4; Sham: *M* = 5.1, SD = 2.2; *p* > 0.05).

### Sham stimulation

We used the Cool B65 A/P unmarked placebo side, which provides a field reduction of ~80%, for sham stimulation. We also delivered a small current pulse to the scalp synchronous to the TMS pulse to replicate the current induced by the TMS pulse in the active condition [29]. Importantly, both the subject and the operator were blind to the condition.

### White noise

The noise was a 40-ms, 103-dB white noise stimulus with an instantaneous rise time delivered via Sennheiser HD280PRO (Sennheiser electronic GmbH & Co., Wedemark, Germany) over-the-ear headphones [30].

### Shock

The shock was a 100 ms, 200 Hz train of stimulation delivered to the right wrist by a constant current stimulator (Digitimer #DS7A, Ft. Lauderdale, FL) via 2, 11 mm disposable Ag/AgCl electrodes (Biopac Item number EL508; Goleta, CA), spaced ~2 cm apart. The shock intensity was determined at the start of the experiment to be at a level that the subjects rated as “uncomfortable but tolerable”.

### Startle habituation

Subjects were exposed to 9, unsignaled presentations of the white noise with a variable inter-noise interval of ~17 s.

### Electromyography

Facial electromyography (EMG) startle responses were recorded from the left orbicularis oculi muscle at 2000 Hz using a Biopac MP160 unit (Biopac; Goleta, CA) via 15 × 20 mm hydrogel coated vinyl electrodes (Rhythmlink #DECUS10026; Columbia, SC).

### Startle

EMG was bandpass filtered from 30 to 300 Hz, rectified, and smoothed using a 20-ms sliding window. Startle responses were scored as the peak (max during the 20 ms to 120 ms post-noise window) – the baseline (50 ms pre-noise window), and converted to *t*-scores (*t*_x_ = [*Z*_x_ × 10] + 50). Noisy trials (baseline SD > 2x run SD) were excluded, and “no blink” (peak < baseline range) trials were coded as 0. To calculate FPS, we subtracted the response during the predictable ITI from the response during the predictable cue. To calculate APS, we subtracted the response during the neutral ITI from the response during the unpredictable ITI. This approach has been traditionally used by our group and was chosen because it allows a direct comparison to other non-NPU threat studies where neutral and predictable blocks are presented without cues (e.g [1, 31]). Another benefit to this approach is that the APS measurement is not contaminated by the cue presentation, which can potentiate the startle response even though it does not carry any predictive information about the shock in the U condition [6].

### Anxiety ratings

Anxiety ratings were continuously recorded throughout the NPU runs, and sampled at the time of delivery of each white noise presentation. These values were then averaged across trials.

### Primary and secondary outcome measures

The primary outcome measure of this study is APS. Secondary outcome measures included FPS, anxiety ratings, BOLD activity during the Sternberg WM task, e-field model estimates, and Accuracy/reaction time during the Sternberg WM task.

FPS was collected as a secondary outcome measure to remain consistent with previous NPU studies [6, 15]. Anxiety ratings were collected to account for placebo effects related to the TMS administration, and to ensure that subjects understood the NPU contingencies. BOLD responses during the Sternberg WM fMRI recordings were assessed to ensure that the Sternberg task activated dlPFC target. Estimates of the current induced in the dlPFC target were recorded to assess the efficacy of the e-field modeling approach. Accuracy and reaction time were collected during the Sternberg WM runs to ensure that subjects were engaged in the task, and to assess the ability of the TMS to influence prefrontal circuits (i.e. target engagement).

## Results

### fMRI data

#### fMRI localization

As a manipulation check, the maintenance period sort > low fMRI difference scores at each subject’s peak location were extracted and submitted to a single-sample t-test against 0 (Fig. 2a). These scores were significantly different from 0 with a large effect size, indicating reliable data for our targeting approach (*t*(18) = 4.495; *p* < 0.001; Cohen’s *d* = 1.059).

#### E-field optimization

To determine the efficacy of the electric-field modeling approach, we calculated the overall effect size achieved by selecting the orientation with the largest normalized e-field estimate. By effect size, we mean the amount of current induced in the target region when using the optimal compared to the least optimal orientation, normalized by the standard deviation of current estimates across orientations. We did this by comparing the optimal orientation to the least optimal orientation. First, we extracted the normalized E-field estimate in the group ROI for the 24 simulations conducted for each subject (Fig. 2b). Then we calculated the effect size achieved from using the yaw vector orientation with the largest normalized E-field estimate (Cohen’s d for orientations = (optimal – least optimal)/SD) for each subject. Across subjects, we observed a moderate effect size (*M* = 0.315; SEM = 0.003) for this comparison.

### Sternberg WM task

To ensure that the Sternberg task was effectively manipulating cognitive load, and to assess target engagement, we performed a 3 (Stimulation: Pre [MRI visit] vs. Active vs. Sham) × 3 (Load: Low, High, Sort) repeated measures ANOVA on the accuracy and RT from the task.

#### Accuracy

For accuracy, we calculated the percent correct for each condition (Table 1). We found significant effects for both load (*f*(2,36) = 19.022; *p* < 0.001; *η*2 = 1.056), and stimulation (f(2,36) = 6.601; p = 0.004; *η*2 = 0.366). We also found a significant load by stimulation interaction (*f*(4,72) = 4.025; *p* = 0.005; *η*^2^ = 0.224). To characterize this effect, we created Active/Sham – Pre-stimulation difference scores, and conducted paired-sample *t*-tests for the low, high, and sort conditions. For low and sort, there was a trend toward a greater improvement for sham compared to active (Low; *t*(18) = −1.766; *p* = 0.094; Cohen’s *d* = 0.402; Sort; *t*(18) = −1.837; *p* = 0.083; Cohen’s *d* = 0.426). For high load, there was a trend in the opposite direction (High; *t*(18) = 1.811; *p* = 0.087; Cohen’s *d* = 0.415).

**Table 1:** Accuracy and reaction time during the Sternberg working memory paradigm.

Load	Low		High		Sort	
	Mean	SD	Mean	SD	Mean	SD
*Percent Correct*						
Pre-Stimulation	87.43	10.79	66.12	14.53	76.61	17.06
Active	86.63	12.89	81.98	15.94	80.39	17.27
Sham	90.57	10.11	73.66	15.67	85.84	15.54
*Reaction Time*						
Pre-Stimulation	1924.82	302.26	2186.27	309.55	2121.65	297.85
Active	2007.36	305.37	2219.40	346.46	2000.72	413.25
Sham	1906.72	374.67	2122.51	417.44	1920.76	448.61

#### Reaction time

For reaction time (Table 1), we observed a main effect for load (*f*(2,36) = 13.084; *p* < 0.001; *η*^2^ = 0.727, and a load by stimulation interaction (*f*(4,72) = 2.665; *p* = 0.039; *η*^2^ = 0.148). For consistency we created Active/Sham – Pre-stimulation difference scores, and conducted paired-sample *t*-tests for the low, high, and sort conditions. Unlike accuracy, we found a trend toward slower reaction times for active stimulation compared to sham in all three conditions (Low; *t*(18) = 1.793; *p* = 0.090; Cohen’s *d* = 0.411, High; *t*(18) = 1.414; *p* = 0.174; Cohen’s *d* = 0.324, Sort; *t*(18) = 1.251; *p* = 0.227; Cohen’s *d* = 0.287).

### NPU

#### Anxiety ratings

To determine whether TMS affected subjective anxiety, we performed a 2 (Timing: Pre vs. Post) × 2 (Stimulation: Active vs. Sham) × 3 (NPU: Neutral vs. Predictable vs. Unpredictable) × 2 (Period: Cue vs. ITI) repeated measures ANOVA on the ratings collected during the NPU task (See Supplementary Fig. 1). We found a main effect of NPU, due to increased anxiety during the threat periods compared to the neutral period (*f*(2,36) = 40.2; *p* < 0.001; *η*^2^ = 2.233). We found a main effect of timing (*f*(1,18) = 8.723; *p* = 0.009; *η*^2^ = 0.485), but no main effect of Stimulation (*p* > 0.05), indicating that subjects reported less anxiety after both active and sham stimulation. However, these main effects were qualified by a timing by period interaction (*f*(1,18) = 22.525; *p* < 0.001; *η*^2^ = 1.25), an NPU × cue interaction (*f*(2,36) = 9.846; *p* < 0.001; *η*^2^ = 0.547), and a timing × NPU × period interaction (*f*(2,36) = 7.027; *p* = 0.003; *η*^2^ = 0.391).

**Fig. 3:**
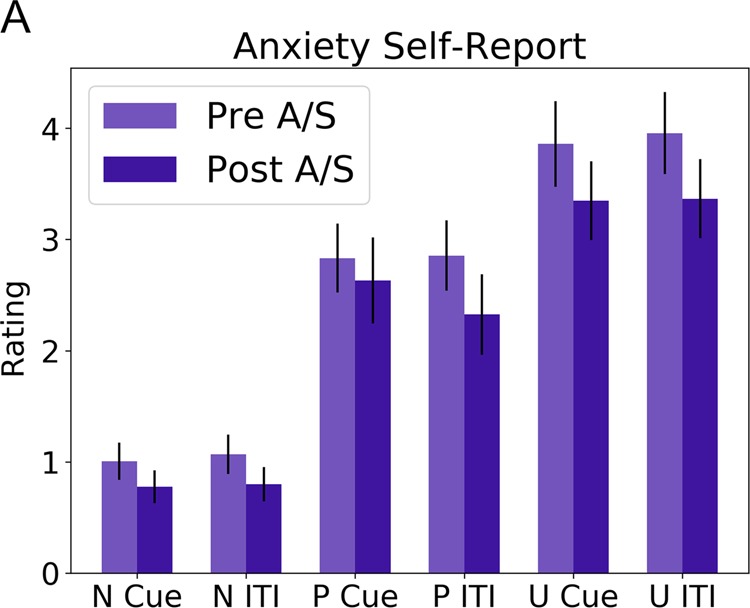
Anxiety ratings during cue and the intertrial interval (ITI) of the Neutral, Predictable, Unpredictable (NPU) threat task. Subjects reported less anxiety after both active and sham stimulation. Scores are averaged across active (A) and sham (S) conditions. Bars indicate Mean ± SEM.

To characterize these interactions, we averaged across stimulation type and conducted paired-sample *t*-tests for the pre vs post stimulation comparison for each NPU × period condition (Fig. 3). Consistent with the main effect of timing, we found significant pre > post differences for the majority of conditions (N Cue; *t*(18) = 2.916; *p* = 0.009; Cohen’s *d* = 0.669; U Cue; *t*(18) = 2.719; *p* = 0.014; Cohen’s *d* = 0.624; N ITI; *t*(18) = 3.215; *p* = 0.005; Cohen’s *d* = 0.738; P ITI; *t*(18) = 2.821; *p* = 0.011; Cohen’s *d* = 0.648; U ITI; *t*(18) = 3.026; *p* = 0.007; Cohen’s *d* = 0.694), with the P Cue period being the exception (*p* > 0.05).

### Startle

**Fig. 4:**
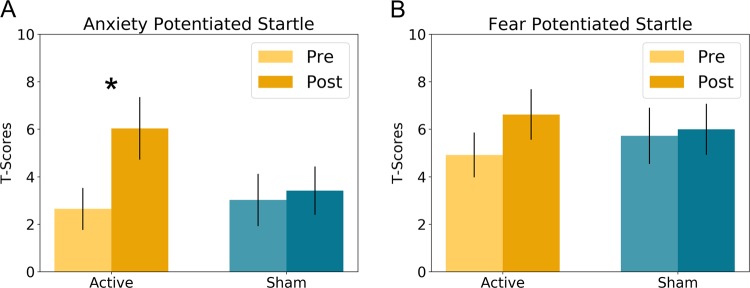
Potentiated startle during the Neutral, Predictable, Unpredictable (NPU) threat task. **a** Anxiety-potentiated startle was significantly increased following active rTMS. **b** Fear-potentiated startle was marginally increased following active rTMS. Bars indicate Mean ± SEM. *p < 0.05.

To quantify APS and FPS, we created the following difference scores (APS = U ITI – N ITI; FPS = P Cue – P ITI; [6, 15] T-scores and raw startle magnitudes for all conditions are plotted in Supplementary Figs. 2 and 3, respectively), clipping outliers to within 2SD of the mean, according to previously published standards [32]. We conducted 2 (Stimulation: Active vs. Sham) by 2 (Timing: Pre vs. Post) repeated measures ANCOVAs on APS and FPS, covarying out ratings of the TMS sensation to account for placebo effects apparent in the NPU rating data. For APS (Fig. 4a), we found a significant Stimulation by Timing interaction (*f*(1,1) = 4.988; *p* = 0.039; *η*^2^ = 1.672). To characterize this interaction, we conducted paired sample *t*-tests for the Pre vs. Post comparison for the active and sham conditions. Counter to our hypothesis, we found a significant increase in APS following active rTMS (*t*(18) = −2.346; *p* = 0.031; Cohen’s *d* = 0.538), while for sham, we found no such effect (*p* > 0.05).

As with APS, for FPS (Fig. 4b) we found a significant Stimulation by Timing interaction (*f*(1,1) = 7.108; *p* = 0.016; *η*^2^ = 1.072), which we characterized using paired sample t-tests for the Pre vs. Post comparison for the active and sham conditions. Like APS, there was an increase in FPS following active stimulation, but this increase was not significant (*t*(18) = −1.092; *p* = 0.289; Cohen’s *d* = 0.251), and there was no effect of sham stimulation (*p* > 0.05).

Because we lowered the stimulation intensity for some of the participants (at their request), we attempted to determine whether this may have impacted APS and FPS. Accordingly, we correlated stimulation intensity (% MT) with the pre-post change in APS and FPS. For both APS and FPS we found moderate, albeit non-significant negative correlations (APS: *r*(18) = −0.273; *p* = 0.258; FPS: *r*(18) = −0.254; *p* = 0.294) with stimulation intensity.

## Discussion

This study investigated the effect of 10 Hz stimulation to the dlPFC on anxiety induced by threat of shock with APS as the primary outcome measure. We hypothesized that APS would be reduced immediately after active rTMS compared to sham. However, contrary to this hypothesis, active rTMS to the dlPFC increased APS. Although additional research is needed to characterize this effect, our results suggest a link between dlPFC and the physiological expression of anxiety. Importantly, the increase in APS is unlikely due to expectancy effects because individuals’ anxiety ratings were reduced after both active and sham stimulation. This novel mechanistic link between the dlPFC and anxious arousal may prove to be an important clinical target for future anxiety treatments.

Given that the findings were counter to our hypothesis, it is important to consider them in light of the other findings in the study. There were two main findings that could be considered contrary to the APS findings. First, although we observed a significant Stimulation by Timing interaction with FPS, unlike APS the follow-up *t*-tests were not significant. These results suggest that the effect of active stimulation on APS may be stronger than the effect of active stimulation on FPS. One possibility for this difference is that the increase in arousal captured by the FPS measure is driven by an acute fear, rather than a sustained anxiety response [15], and, as we have shown previously [18, 33] the former is less susceptible to various types of experimental manipulation than the former. However, the most likely explanation for these results is that subjects expected the TMS to reduce their anxiety, and so they reported less anxiety. This dissociation between implicit (APS) and explicit (ratings) arousal is well documented in the emotional learning field (e.g. LeDoux et al. [34]).

Our hypothesis for decreased APS with 10 Hz TMS to the dlPFC was based on the assumptions that (1) the dlPFC is involved in anxiety down-regulation, and (2) 10 Hz rTMS would facilitate this down-regulation. In the next sections, we will discuss the evidence for and against these assumptions. In the Future Directions section, we will discuss the follow-up work needed to further test these assumptions.

### Top-down regulation

Although the finding of increased APS following active 10 but not sham Hz rTMS to the right dlPFC was counter to our hypothesis, it is consistent with the frontal asymmetry hypothesis relating to negative affect [35, 36]. According to this hypothesis, right prefrontal excitability (i.e. decreases in right prefrontal alpha) [9], may be associated with negative affect and high arousal, while left prefrontal excitability may be associated with positive affect [35, 36]. This hypothesis, form the basis for left dlPFC-focused TMS treatments for depression that have been largely successful [37]. For instance, inhibitory continuous theta burst stimulation (cTBS) to the right dlPFC leads to accelerated habituation of the overall startle response during affective picture viewing [38], and cTBS to the left dlPFC attenuates positive affective startle modulation [39]. Similarly, high-frequency rTMS to the right dlPFC increases threat-related attentional capture in those with high trait anxiety [40]. In line with this thinking, there have been efforts to extend this model to treat anxiety by reducing right dlPFC activity with low-frequency stimulation, which has led to some success [8]. One possible explanation for this concerns the distinction between anxiety expression and anxiety regulation.

The above asymmetry model is based on the observation of elevated right dlPFC activity accompanies high anxiety, and assumes that this activity mediates anxiety expression [41]. However, it is also possible that this right dlPFC activity is more involved in top-down regulation [5, 6], which was the basis for the current study. Indeed, there is some evidence from neuromodulatory studies suggesting that exciting the right dlPFC produces anxiolytic effects, while inhibiting the right dlPFC produces the opposite effect. For instance, low-frequency rTMS to the right dlPFC increases heart rate during an affective picture viewing task [42]. Similarly, high-frequency stimulation to the right dlPFC reduces amygdala responses to negative faces [43], and single pulses to the right dlPFC reduce amygdala activity during simultaneous TMS/fMRI and PTSD patients [44].

Indeed, both the expression and regulation hypotheses are consistent with our previous data showing a positive relationship between right dlPFC activity and anxiety. According to the expression hypothesis, right dlPFC activity increases may be related to increases in anxiety symptoms [45, 46]. In contrast, according to the regulation hypothesis, anxiety increases drive activity in expression-related regions (e.g. the amygdala, BNST, etc.) [47], which engage down-regulation processes that drive activity in the right dlPFC [5, 6]. This is consistent with the broader role of the dlPFC in emotion regulation and distractor suppression in general. Our current results are consistent with the expression hypothesis and by extension the frontal asymmetry hypothesis; however, it should also be mentioned that we did not directly test the effects of left vs. right dlPFC stimulation, so future work is needed to test the laterality of this effect. In contrast, assuming excitatory effects from the 10 Hz stimulation protocol, our results are not consistent with the regulation hypothesis.

### Frequency-specific effects of rTMS

Although the goal of this study was to experimentally test this top-down inhibition hypothesis by experimentally increasing dlPFC excitability and measuring the effect on anxious arousal, our hypothesis depended on the assumption that 10 Hz rTMS would increase right dlPFC activity post stimulation, with few off-target effects [48, 49]. Indeed, there is evidence that high-frequency stimulation increases cortical excitability both within-session [49], and acutely after the stimulation session [48]. High-frequency stimulation has also been shown to be effective at treating depression when delivered to the left dlPFC [37], whereas anxiolytic effects have been reported with 1 Hz to the right dlPFC [8]. However, it should be noted that the after-effects of high-frequency rTMS are thought to be highly parameter dependent, varying also as a function of duration and intensity, with lower intensities often leading to decreases in excitability post stimulation [50]. One possible explanation for our increase in APS following active stimulation may be our decision to decrease the stimulation intensity when requested by the subjects. Indeed, we do observe a moderate, albeit non-significant, negative correlation between stimulation intensity (as a percentage of motor threshold) and the pre-post change in APS (*r* = −0.27). We understand that this intensity adjustment is not common practice; however, our shock threat manipulation may have sensitized our subjects to pain. In addition, given the nature of our study, anxiety manipulation through threat of an unpleasant shock, it was important that the rTMS not be perceived as more unpleasant than the shock itself, which is indeed the case (Unpleasantness Ratings: shock = 8.84; active rTMS = 6.26). It is also important to note that the rTMS was done offline, so that anticipation of the rTMS did not contaminate the measures collected during the NPU task.

### Sternberg performance

The current study included the Sternberg WM task, which was used both as a functional localizer as well as a method to control the subjective state of the subject during the stimulation sessions. Although this study was not designed to directly test the effects of rTMS to the right dlPFC on Sternberg performance, we did observe two potential differences as a function of stimulation type. First, people tended to be marginally slower during active compared to sham. Second, practice effects for accuracy (i.e. changes from pre-stimulation baseline) tended to differ as a function of working memory load, with practice effects being marginally larger for sham compared to active for low and sort, while practice effects being marginally larger for active compared to sham for high. These results are consistent with previous work showing that targeting WM circuits can lead to load-related effects on WM performance [28, 51]. However, these results should be considered with caution for two reasons. First, the Sternberg WM task was included in the study design to facilitate the effects of TMS on anxiety, and so it was not optimally designed to measure the effect of TMS on WM performance. Second, the effects observed were marginal, and only included to show the degree to which the rTMS targeted WM-related circuitry.

### Future directions

The broader goals of this line of research are to identify mechanisms underlying the expression and regulation of anxiety, and to develop potential new treatments. By uncovering a link between right dlPFC activity and APS modulation, the current work represents a first step in this process. Again, it is important to note that additional work targeting both the left and the right dlPFC is needed to confirm the laterality of these findings. Given the existing work using low-frequency rTMS to treat anxiety symptoms in depression [8], it is tempting to conclude that this link primarily reflects a role for the right dlPFC in anxiety expression. However, given the large TMS parameter space [52], and parameter-dependent nature of rTMS after-effects [50], this conclusion may be premature. Future work should focus on characterizing the nature of this link in terms of expression-related vs. regulation-related processing. One key component to this work will be to systematically explore the relationship between stimulation frequency and intensity on anxiety expression within-session. Another key component will be to explore plasticity-related after-effects of rTMS on anxiety by extending the interval between the rTMS session and the test session. Because potential future treatment effects will need to be long-lasting, this latter component will also be an important step in developing robust treatment options.

### Strengths and limitations

There were several strengths to this study. The primary strength being the novel use of APS as an outcome measure to assess the effect of rTMS. Importantly, this physiological measure allowed us to measure the objective effect of the active stimulation protocol on anxiety [53], independent of the subjective placebo effects induced by both the active and sham stimulation. This is an important step because anxiety is highly dimensional, so this healthy volunteer approach employing APS could be used from proof-of-concept in the development of novel rTMS treatment strategies [54]. Although it is out of the scope of the current work to directly test the predictive validity of APS as predictive of clinical outcomes, there is ample evidence to suggest that APS is influenced by many of the same therapeutic interventions as clinical anxiety [18, 55], and is elevated in women, who are known to suffer from anxiety disorders at higher rates than men [56]. Both the operator and the subject were blinded to the TMS condition, and the subjects were given a realistic electric stimulation sham to prevent unblinding [29]. Another strength is that we used objective methods to obtain a priori criteria to determine the site (fMRI-based localization), orientation (E-field modeling; [26]), and subjective state of the subject during stimulation (Sternberg WM maintenance; [12, 57]), thereby reducing the experimenter degrees of freedom inherent in the high dimensionality parameter space of rTMS.

The primary limitation of the current work is that we measured anxiety immediately post active or sham rTMS, making it difficult to assess how changes in right dlPFC plasticity affected anxiety independent of the acute effects of the rTMS on right dlPFC excitability [58]. Another limitation is that although the operator was blinded to the rTMS condition, the research assistant had to remain unblinded, in order to set the coil (active vs. sham) and e-stim intensity. However, it should be noted that the research assistant had minimal patient contact during the visit. Another limitation is that we did not formally record participant guesses for the sham/real condition. However, these limitations are not likely sufficient to explain the increase in APS following active stimulation, because both our initial hypotheses (i.e. experimenter bias) and the subjective anxiety ratings (i.e. participant’s expectations) run counter to the APS finding. One final limitation of the current study is the relatively small N of the current work. Our initial power analysis suggested that we would need 26 subjects; however, due to time and resource constraints we were only able to collect 24 (19 included in the final analysis). Future studies should be conducted with larger sample sizes to replicate these findings.

## Conclusions

We found that 10 Hz rTMS to the right dlPFC increased anxiety potentiated startle in healthy volunteers, providing a novel link between right dlPFC activity and the physiological expression of anxiety. Although the direction of the effect was counter to our hypothesis, it is consistent with current models of prefrontal asymmetry. Future work should expand on these findings by testing for frequency and amplitude specific effects, and by extending the stimulation-test interval to periods outside the consolidation window (i.e. > 24 h).

## Funding and disclosure

This project was supported in part by a 2018 NARSAD Young Investigator Grant from the Brain & Behavior Foundation (NLB). Financial support of this study was provided by the Intramural Research Program of the National Institute of Mental Health, ZIAMH002798 (ClinicalTrial.gov Identifier: NCT03027414: Protocol ID 17-M-0042). The authors declare no competing interests.

## Supplementary information

CONSORT flow diagram

Supplemental Material
