# Supplementary material for: Mechanistic link between right prefrontal cortical activity and anxious arousal revealed using transcranial magnetic stimulation in healthy subjects
Source: Neuropsychopharmacology. 2019 Dec 2;45(4):694–702. doi: 10.1038/s41386-019-0583-5 (PMC7021903; doi:10.1038/s41386-019-0583-5)
Supplement: Supplementary file 1 — CONSORT flow diagram [file 41386_2019_583_MOESM1_ESM.docx]

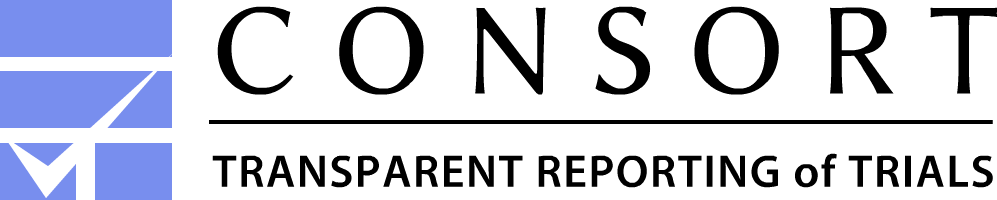


**CONSORT 2010 Flow Diagram**

## Analysis

Allocated to intervention (n= 24)

♦ Received allocated intervention (n= 19)

♦ Did not receive allocated intervention (give reasons) (n= 5) discomfort associated with TMS, 2 subjects; 1 subject; scheduling conflict, 1 subject; incompatible hairstyle, 1 subject dizziness

Analysed (n= 19)
♦ Excluded from analysis (give reasons) (n= 0)

## Enrollment

## Allocation

Randomized (n= 24)

Assessed for eligibility (n= 24)
