# Supplementary material for: Mechanistic link between right prefrontal cortical activity and anxious arousal revealed using transcranial magnetic stimulation in healthy subjects
Source: Neuropsychopharmacology. 2019 Dec 2;45(4):694–702. doi: 10.1038/s41386-019-0583-5 (PMC7021903; doi:10.1038/s41386-019-0583-5)
Supplement: Supplementary file 2 — Supplemental Material [file 41386_2019_583_MOESM2_ESM.docx]

**
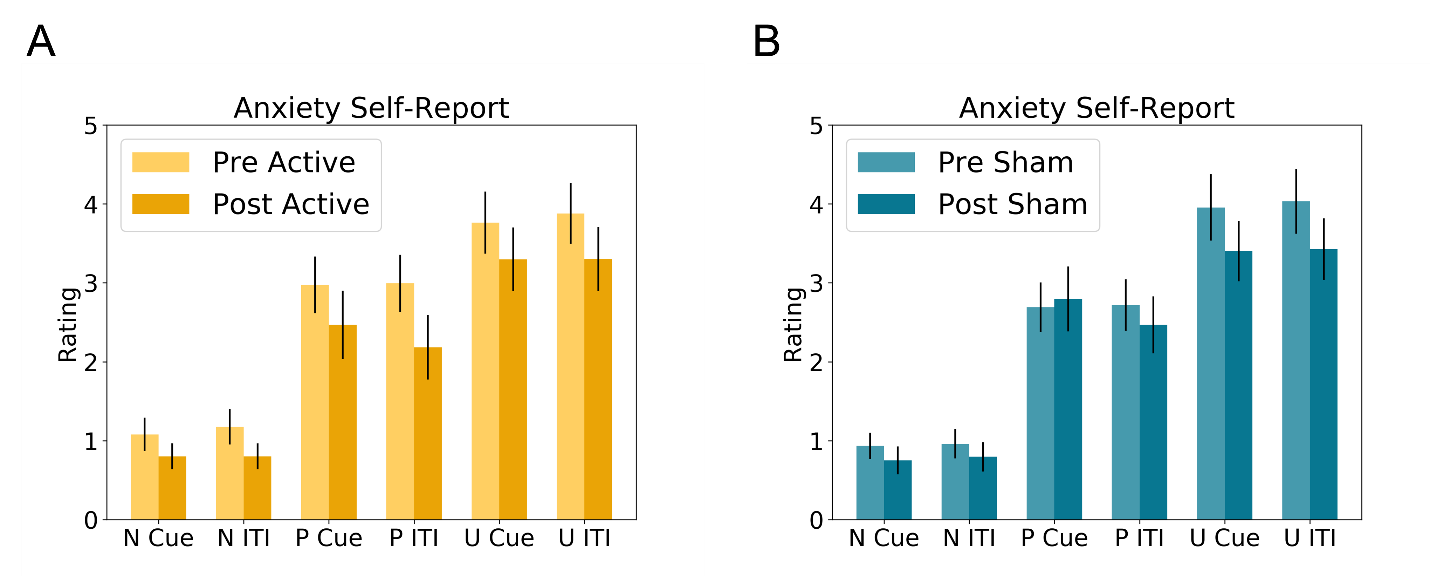
**

**Supplemental Figure 1: Anxiety ratings during cue and the intertrial interval (ITI) of the Neutral, Predictable, Unpredictable (NPU) threat task.** Bars indicate Mean +/- SEM.

**
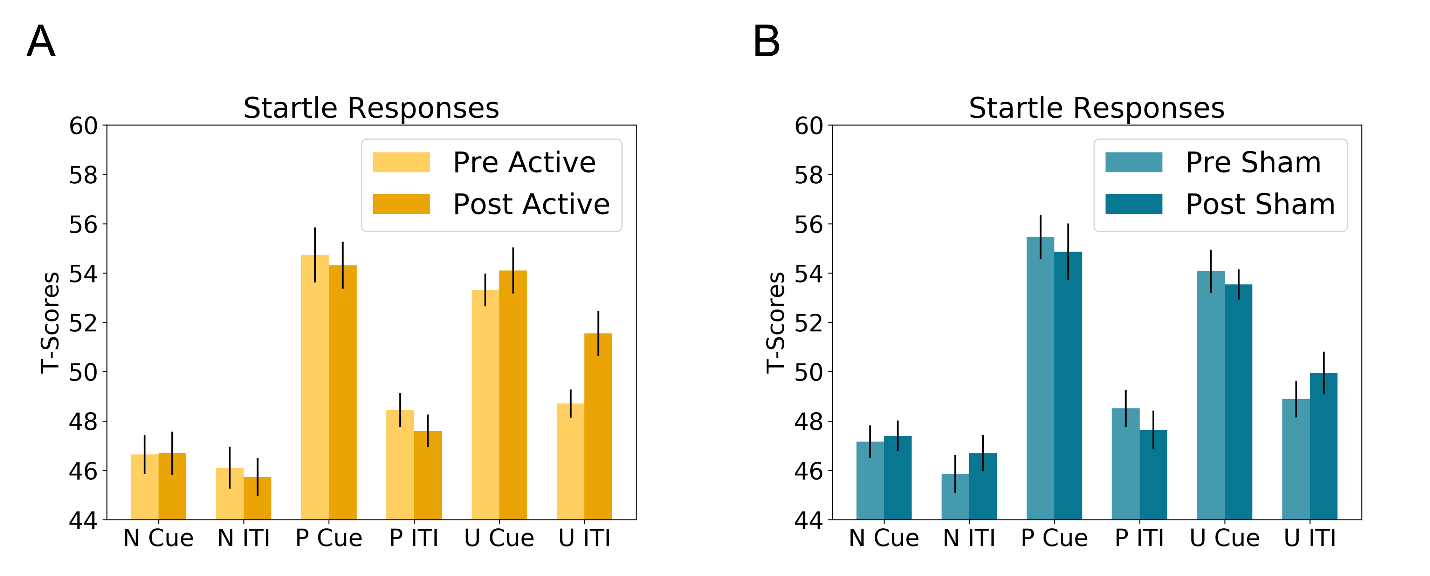
**

**Supplemental Figure 2: Startle data (T-scores) during cue and the intertrial interval (ITI) of the Neutral, Predictable, Unpredictable (NPU) threat task.** Bars indicate Mean +/- SEM.

**
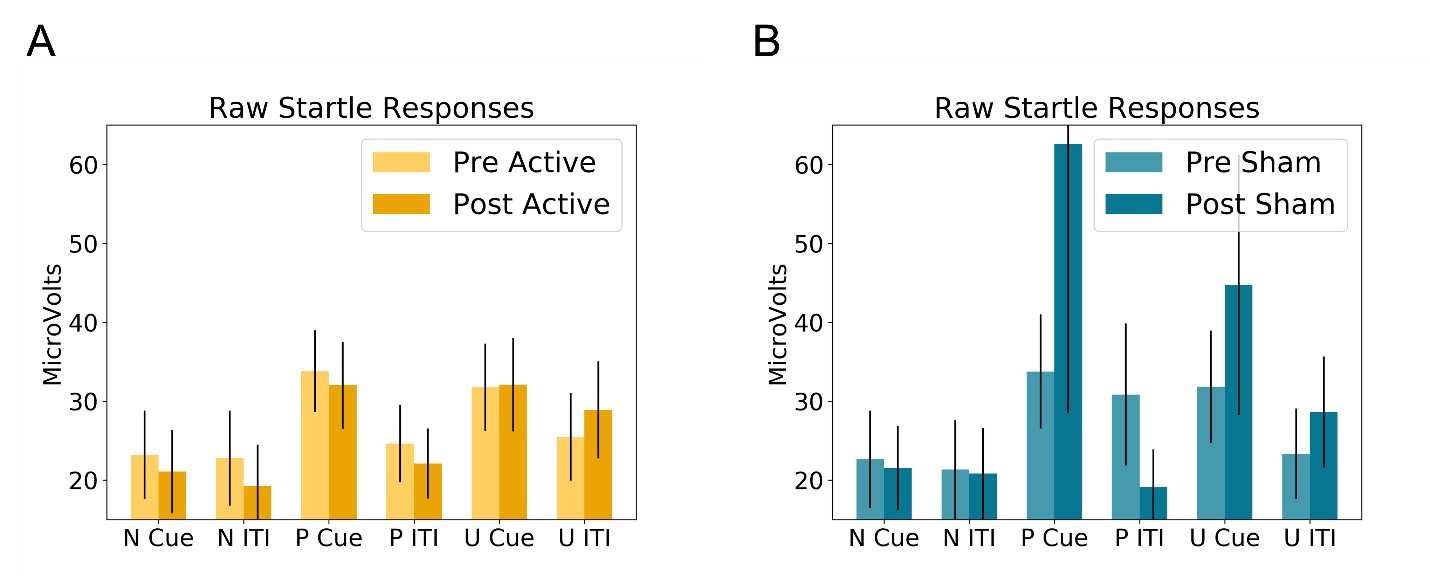
**

**Supplemental Figure 3: Startle data (raw) during cue and the intertrial interval (ITI) of the Neutral, Predictable, Unpredictable (NPU) threat task.** Bars indicate Mean +/- SEM.
